# SOCIAL ENGAGEMENT IN LATER LIFE: INTERDEPENDENCE OF MARRIED COUPLE’S MENTAL HEALTH WELL-BEING

**DOI:** 10.1093/geroni/igac059.2864

**Published:** 2022-12-20

**Authors:** Yulri Kim

**Affiliations:** Iowa State University, Ames, Iowa, United States

## Abstract

Social engagement in later life often provides a sense of belongings and social identity. Given its salient meanings for older adults in Korea where social ties play important roles on the mental health and well-being, different conceptualizations of social engagement have been examined, such as formal and informal social activities. As numerous studies have suggested, informal activities are associated with better well-being, however, it is unclear whether formal social engagement of husbands and wives influence the other partner’s mental health outcomes. Based on the Interdependence Theory (Rusbult & Van Range, 2008), we expect that formal and informal social engagement of husbands and wives are associated with their own depressive symptoms and further those of their spouses. Using a sample of 1,195 couples (Nf 2,390) of married older adults aged 65 and above from the 8th wave of Korean Longitudinal Study of Aging (KLoSA), we tested the relationship between social engagement and depressive symptoms using actor partner interdependence model (APIM). The findings indicated that for both husbands and wives, there was a negatively significant association between one’s informal social activity on their own depressive symptoms (actor effect) and their spouse’s depressive symptoms (partner effect). No significant findings were observed for formal social activities. Overall, this study suggests the importance of informal social activity compared to formal social activity to lower depressive symptoms for themselves and their spouses in later life. Moving forward, incorporating a partner’s informal social activity can provide useful information in predicting one’s own mental health well-being.

